# Super-resolution microscopy in studying neuroendocrine cell function

**DOI:** 10.3389/fnins.2013.00222

**Published:** 2013-11-25

**Authors:** Anneka Bost, Mathias Pasche, Claudia Schirra, Ute Becherer

**Affiliations:** ^1^Physiologisches Institut, Universität des SaarlandesHomburg/Saar, Germany; ^2^Division of Neurobiology, MRC Laboratory of Molecular BiologyCambridge, UK

**Keywords:** chromaffin cell, membrane capacitance, amperometry, TIRFM, SIM, PALM, STORM, STED

## Abstract

The last two decades have seen a tremendous development in high resolution microscopy techniques giving rise to acronyms such as TIRFM, SIM, PALM, STORM, and STED. The goal of all these techniques is to overcome the physical resolution barrier of light microscopy in order to resolve precise protein localization and possibly their interaction in cells. Neuroendocrine cell function is to secrete hormones and peptides on demand. This fine-tuned multi-step process is mediated by a large array of proteins. Here, we review the new microscopy techniques used to obtain high resolution and how they have been applied to increase our knowledge of the molecular mechanisms involved in neuroendocrine cell secretion. Further the limitations of these methods are discussed and insights in possible new applications are provided.

## Introduction

**Figure 1:**
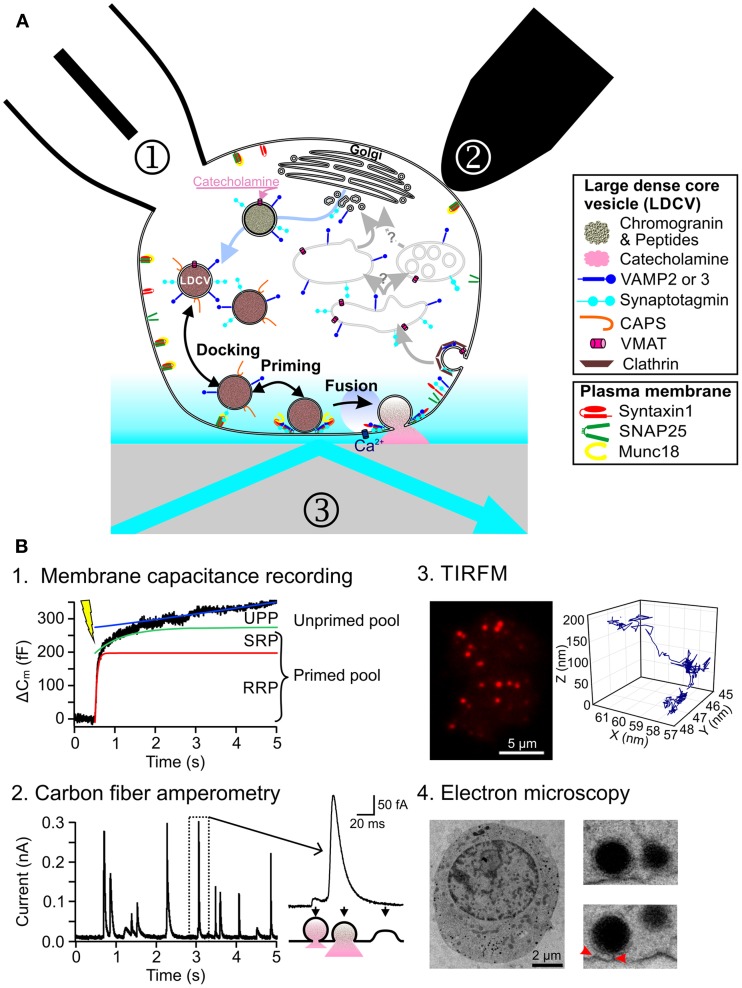
**Current methods to study regulated exocytosis in chromaffin cells. (A)** Model of regulated exocytosis in chromaffin cells depicting a number of proteins involved in the process. Exocytosis of LDCVs can be monitored by ① membrane capacitance through patch clamp electrophysiology, ② carbon fiber amperometry and ③ TIRFM. **(B)** Example of exocytosis measurement in chromaffin cells. (B1) Representative trace of a membrane capacitance recording in which exocytosis was induced by flash photolysis of caged Ca^2+^. Three functional pools can be determined by fitting the data with 3 components: a fast exponential (RRP, red line), a slow exponential (SRP, green line) and a linear regression (UPP, blue line). (B2) Release of catecholamines from single LDCVs can be observed as individual spikes by carbon fiber amperometry (left). The spike shape provides information about the fusion pore opening (right). (B3) TIRFM picture of a bovine chromaffin cell expressing NPY-mCherry (left). Due to the high signal to noise ratio, the LDCVs can easily be seen as individual spots. They can be tracked over time thus revealing a complex behavior (right). (B4) Electron micrograph of an embryonic mouse chromaffin cell, fixed using high-pressure freezing method (left). The close up of some LDCVs shows astounding morphological details (right). Red arrows indicate fine tethers that appear to bridge the LDCV and the plasma membrane.

The main function of neuroendocrine cells, e.g., chromaffin cells, is the regulated release of hormones or peptides into the blood stream. This function is well documented in several reviews (Becherer and Rettig, 2006; Stevens et al., 2011; Jahn and Fasshauer, 2012; Kasai et al., 2012). Briefly, regulated exocytosis is a multi-step process controlled by calcium (Figure 1A). In order to fuse, large dense core vesicles (LDCVs) containing catecholamines approach the plasma membrane (PM) along actin filaments (Villanueva et al., 2012). They dock to the PM to the target-SNARE (t-SNARE) acceptor complex composed of syntaxin1 and SNAP-25, via synaptotagmin (de Wit, 2010). This process is controlled by Munc18 which acts at several steps during exocytosis (Rizo and Sudhof, 2012). After docking LDCVs undergo maturation reactions in which the vesicular SNAREs, vesicle associated membrane protein 2 and 3, also called synaptobrevin and cellubrevin, associate with the t-SNAREs to form the SNARE core complex. This reaction, that stably binds LDCVs to the PM, is regulated by a variety of proteins such as Ca^2+^-dependent activator protein for secretion (CAPS), complexin, snapin or tomosyn (Becherer and Rettig, 2006). Upon increase of Ca^2+^ above a concentration of 0.5–0.9 μM, fusion is initiated by the interaction of synaptotagmin with the SNARE complex and the PM (Sudhof, 2012). Proteins such as complexin control this reaction. After exocytosis, the LDCV membrane and protein components are taken up via clathrin dependent endocytosis and processed through poorly understood recycling (Becherer et al., 2012). The development of this rather complex model of the exocytosic pathway was enabled by an array of innovative measurement methods that were applied to neuroendocrine cells.

## Methods to study chromaffin cell function

In neuroendocrine cells, regulated exocytosis has been studied since the late 1960s using biochemical methods (Schneider et al., 1967). Among other important findings, researchers were able to describe the Ca^2+^-dependency of exocytosis (Pollard et al., 1982). The pace of research in this field greatly picked up with the launch of high time resolution measurement methods. In 1982 using patch-clamp electrophysiology, Neher and Marty (1982) were able to measure changes in the membrane capacitance of chromaffin cells that corresponded to either the addition (exocytosis) or subtraction (endocytosis) of vesicular membrane to the PM. Using complex depolarization protocols (Gillis et al., 1996; Voets et al., 1999) or flash photolysis of caged Ca^2+^ (Kaplan and Ellis-Davies, 1988; Neher and Zucker, 1993) it was possible to dissect four main functional pools of LDCVs termed reserve pool, unprimed pool (UPP), slowly releasable pool (SRP) and readily releasable pool (RRP) (Figure 1B1). It was hypothesized that LDCVs dock to join the UPP and that while priming occurs, LDCVs proceed in a sequential manner through SRP and RRP (Sorensen, 2004). The molecular machinery mediating these reactions was dissected using gain or loss of function assays (Becherer and Rettig, 2006). Ten years after establishing membrane capacitance recording, carbon fiber amperometry was developed (Wightman et al., 1991). With this technique the release of oxidizable neurotransmitters or hormones such as catecholamines from individual LDCVs can be measured with very high temporal resolution (Bruns and Jahn, 2002). The derived kinetics of release were used to identify proteins such as synaptobrevin, that play a role in fusion pore opening during exocytosis (Borisovska et al., 2005) (Figure 1B2). By combining carbon fiber amperometry with membrane capacitance recording, it was then possible to discriminate between exo- and endocytosis when they occur simultaneously (Borges et al., 2008). Furthermore, this combination was instrumental in uncovering the role of for example CAPS in loading LDCVs with catecholamines (Speidel et al., 2005).

The main limitation of both methods is that they measure directly the very last step of exocytosis and they provide only little information about docking. Electron microscopy (EM) is therefore often used to complement this information (Ashery et al., 2000; Yizhar et al., 2004; de Wit et al., 2009). Vesicles are considered morphologically docked if they are touching the PM or located within 30 nm distance to the PM (Verhage and Sorensen, 2008; de Wit, 2010). These vesicles can belong to all three functional pools UPP, SRP or RRP. The development of a fixation method involving high-pressure freezing produced nearly artifact free, highly preserved cell morphology (Studer et al., 2001; Vanhecke et al., 2008). Electron micrographs of chromaffin cells fixed with this method, revealed fine structures at the contact regions between LDCVs and the PM, which might correspond to assembled SNARE proteins (Figure 1B4). Due to this increased resolution a new morphological classification was introduced of docked and tethered vesicles (Verhage and Sorensen, 2008). Until now it was not possible to clearly associate a functional pool to those two morphologically different pools of LDCVs, thereby generating quite some confusion in the field.

In the 1990s total internal reflection fluorescence microscopy (TIRFM) was first introduced in the field of exocytosis (Axelrod, 1981; Steyer et al., 1997; Oheim et al., 1998; Oheim and Stuhmer, 2000). It is used to visualize individual LDCVs approaching the PM and their fusion. The principal feature of TIRFM is that a thin evanescent field of light with decaying exponential excitation energy is generated at the interface of the glass coverslip and the cell. Thus, excitation of fluorophores is restricted to a shallow layer close to the PM (Figure 1B3). This technique provides an axial (z) resolution well below 100 nm, while the lateral (x, y) resolution is about 250 nm. The highly contrasted pictures of individual fluorescently labeled LDCVs and the possibility to follow them over time raised very high expectations that TIRFM would provide profound insights in the molecular machinery of docking and priming (Steyer et al., 1997; Oheim and Stuhmer, 2000; Johns et al., 2001). This turned out to be much more complex than originally anticipated (Oheim and Stuhmer, 2000; Nofal et al., 2007). TIRFM helped to understand the role of Munc18-1 (Toonen et al., 2006) and Ca^2+^ (Pasche et al., 2012) during docking, and the function of tomosyn in priming (Yizhar and Ashery, 2008).

Taken together, using a combination of membrane capacitance recording, carbon fiber amperometry and EM very effectively uncovered the function of several proteins, such as the SNARE proteins or Munc13, which play a role at only one step of exocytosis in chromaffin cells. TIRFM helped to examine the function of certain proteins or substances, such as Munc18 or Ca^2+^, that mediate several steps of exocytosis. However, the results of these studies become more and more complex to interpret as we investigate the role of proteins that appear to be involved throughout exocytosis, e.g., Synaptotagmin. The question is whether super-resolution microscopy can help in this quest by providing a link between morphological (EM) and functional data (membrane capacitance recording, carbon fiber amperometry or TIRFM).

## Super-resolution microscopy

The aim of super-resolution microscopy is to provide similar resolution as EM but with light microscopy. In light microscopy the wave-like nature of light limits spatial resolution to half the wavelength of the observed light. This so called diffraction barrier was established by Ernst Abbé 140 years ago and is expressed by the formula:

$$d=\frac{\lambda }{2(n\sin\theta )}$$

(with d the diameter of the spot generated by light of the wavelength λ that travels in a medium with refractive index *n* and converges with an angle θ).

All microscopy technologies developed at the end of the twentieth century, such as confocal microscopy or TIRFM, improve the signal-to-noise ratio and thus produced highly contrasted and crisp images that showed a wealth of unprecedented details, but the resolution was still diffraction limited. An early approach was to use deconvolution algorithms to subtract the predicted point spread function (PSF) of individual fluorophores from the image and thus to reduce the contribution of diffracted light. This technique produces images with even better signal-to-noise ratio, but the resolution improvement is modest. Furthermore, due to the use of flawed PSFs, this technique is prone to generate artifacts like non existing signal patterns. The new super-resolution microscopy methods have addressed most of these problems and can achieve a resolution down to 10 nm (Hell, 2009; Dani and Huang, 2010; Tonnesen and Nagerl, 2013a).

## Structured illumination microscopy (SIM)

**Figure 2:**
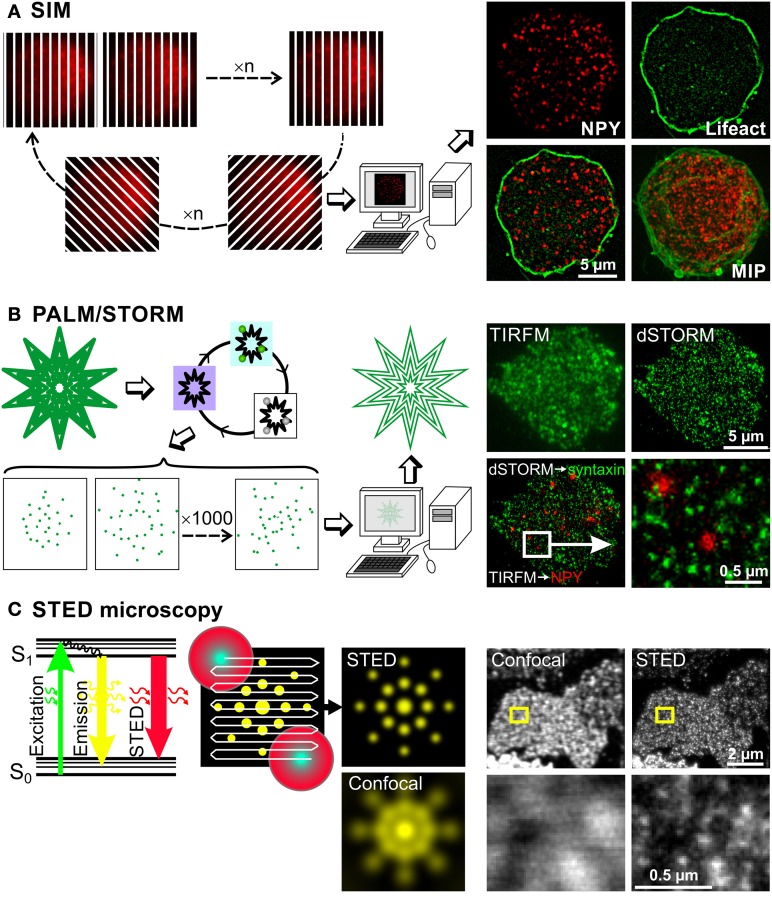
**Super-resolution microscopy methods to investigate chromaffin cell function**. Basic principles of three super-resolution microscopy methods are depicted on the left, while some exemplary images using the respective technique are shown on the right. **(A)** In SIM microscopy a stripe pattern of light, which is shifted and rotated, is applied to the cell, so that the entire cell is illuminated within several images. These images contain sub-diffraction-limited structural information, which is extracted via computer processing using Fourier transformations. Resulting images have a lateral and axial resolution of 100 and 300 nm, respectively. This can be appreciated on images of a bovine chromaffin cell, expressing NPY-mCherry and Lifeact-GFP labeling LDCVs and the F-actin, respectively. MIP: maximum intensity projection. **(B)** The principle of PALM and STORM exploit the properties of certain fluorophores that can be switched on and off. At first, all the fluorophores are pushed in a metastable dark state by illuminating them with their specific excitation light. Then, few molecules are brought back to the ground state using a mild illumination at usually 405 nm and visualized using their excitation light, which switches them off again. These cycles are repeated up to 10,000 times generating a movie of blinking fluorophores. The labeled structure is then reconstituted by plotting their exact calculated position. This method generates images with lateral resolution of 10 to 30 nm. This is shown on pictures of a bovine chromaffin cell. The LDCVs were marked by NPY-mCherry overexpression via Semliki Forest virus (shown in red) and the native syntaxin1 (shown in green) was labeled via monoclonal antibody (Synaptic Systems GmbH) and Alexa 647 anti-mouse secondary antibody (Invitrogen). The gain of resolution can clearly be appreciated by comparing the pictures of syntaxin1 acquired with TIRFM and the picture of the same cell acquired with dSTORM. Due to dSTORM resolution, we observed that LDCVs were usually not located on syntaxin1 clusters. **(C)** As can be seen on the Jablonski diagram, the energy of an excited fluorescent molecule can be completely depleted by a photon that matches the energy difference between its excited (S1) and the ground electronic state (S0) before spontaneous fluorescence emission occurs. This process effectively depletes the S1 state of a fluorescent molecule by using a depleting laser that has high photon density, and a higher wavelength than the emission wavelength of the fluorophore. In this example a fluorescent protein such as YFP is excited at 514 nm and releases its fluorescent light at around 520 nm. The stimulated emitted photons are not visible for the light detector (photomultiplier tube, avalanche photodiode) as they travel in the same direction as the stimulating laser beam but good emission filters are needed to block scattered light. Using this technique a STED beam consisting of a beam at the excitation wavelength surrounded by a donut shaped red light beam is applied to the probe. Normal emission occurs only from the central spot thus defining the size of the measured voxel. The STED beam scans the entire probe and the super resolved image is generated online. The lateral resolution is generally 20–50 nm. This very high resolution can be appreciated on the images taken from Sieber et al. (2006) in which syntaxin1 was visualized using antibody labeling on membrane sheets generated from syntaxin1 overexpressing PC12 cells. Images in the lower row correspond to a magnification of the small yellow square drawn on the images in the upper row.

SIM achieves super-resolution by extracting fine structural details from the interference of a structure with predetermined illumination patterns. When a periodic illumination pattern, such as stripes, is applied to a fluorescent sample, an interference pattern is generated. The diffraction-limited fringes of this interference pattern, called moiré fringes, contain information about underlying structural pattern of the sample that cannot be observed with conventional light microscopy. By shifting and rotating the illumination pattern, sub-diffraction-limited structural information of the sample can be extracted from Fourier transformations of the resulting interference pattern (Gustafsson, 2000; Heintzmann et al., 2002) (Figure 2A). This produces a doubling of both lateral and axial resolution reaching 100 and 300 nm, respectively. The resolution of the calculated image depends on the number of unique raw images acquired with different diffraction patterns/orientations. In order to generate a single plane highly resolved image a minimum of 15 different illumination patterns have to be applied. Currently, the minimum time required for this acquisition is about 300 ms for a single plane and about 8 s for 7 μm thick chromaffin cells (Shao et al., 2011). This time frame is incompatible for life imaging of fast moving structures like LDCVs. However, it can easily be used to visualize 3D distribution of LDCVs in fixed cells without the need of lengthy procedures used in EM. Furthermore, it allows performing precise colocalization studies (Fiolka et al., 2012) in immune cells (Brown et al., 2011; Pattu et al., 2011; Matti et al., 2013) and neurons (Pielage et al., 2008; Sheets et al., 2012; Khuong et al., 2013) that might help to uncover interactions between proteins.

## Photoactivated localization microscopy (PALM) and stochastic optical reconstruction microscopy (STORM)

In PALM or STORM the resolution has been improved to much greater extent since they provide a lateral resolution of 10–30 nm and about 50 nm axial resolution (Betzig et al., 2006; Hess et al., 2006; Rust et al., 2006; Huang et al., 2008; Tatavarty et al., 2009; Shim et al., 2012). Both PALM and STORM rely on the property of certain fluorophores that can be photoactivated and switched on and off alternately (Folling et al., 2008; Shim et al., 2012). PALM relies on photoactivated fluorescent proteins (Betzig et al., 2006; Hess et al., 2006) while STORM, also called direct STORM (dSTORM), was developed using cyanide dyes (Bates et al., 2005; Rust et al., 2006; Heilemann et al., 2008). To generate super-resolved images, the fluorescence of the sample is first entirely quenched with the normal excitation wavelength of the fluorophore, converting it from a ground state to a metastable dark state. Then fluorescence is slowly reactivated, either by itself or by a mild illumination with light at 405 nm (Figure 2B). Importantly, not all the fluorescence is reactivated simultaneously, instead individual fluorophores are activated stochastically over time. They are visualized by the normal excitation light, which also brings them back in the dark state. A movie of blinking fluorescent molecules is then acquired over several minutes. Their position can be precisely determined using a simple Gaussian fit algorithm, as long as their fluorescent signals do not overlap. The quality of the position determination and thus of the final image resolution depends on the signal-to-noise ratio of the original image, on the internal jittering of the microscope and on the labeling density of the marked structure. The super-resolution image is then reconstructed by plotting the peak position of all blinking molecules. Depending on the sample size and the staining quality, 100 to 10,000 images have to be acquired to reconstruct one single image. Due to the large number of images that need to be recorded and their lengthy processing, this technique is not applicable to life cell imaging. However, PALM was recently used to follow single fluorescent proteins over time in living cells (Tatavarty et al., 2009; Izeddin et al., 2011; Sochacki et al., 2012). When comparing PALM to STORM, both produce images with similar resolution although STORM relies on antibody labeling which increases the length between the protein of interest and the fluorescent label (but see Ries et al., 2012). Furthermore, in STORM a toxic reducing buffer is used to reactivate the cyanide fluorophore (but see Klein et al., 2011). On the other hand, PALM relies on the overexpression of proteins tagged to individual fluorescent proteins, thus overexpression artifacts can occur. In brief, despite their respective weakness, PALM and STORM clearly close the gap between EM and light microscopy. They allow very precise localization of proteins in the cell without the need of complex immunogold techniques (Mennella et al., 2012; Macgillavry et al., 2013).

## Stimulated emission depletion (STED) microscopy

In contrast to the aforementioned super-resolution microscopy techniques, STED microscopy does not rely on post processing of blurry raw images but rather uses a specific illumination method and photo-physics to generate directly highly resolved images. In STED microscopy the excited fluorescent dye molecules return to the ground state (S0, Figure 2C) via the process of stimulated emission, which is induced by a STED laser beam. The wavelength of the STED beam is at the tail end of the emission spectrum of the dye, where it does not excite the dye and where it can be spectrally separated from the spontaneous fluorescence. The fluorescence quenching scales with the intensity of the STED laser beam. The trick is to focus a depletion laser into a donut shape and superimpose this onto the focused laser excitation spot (Figure 2C middle). The spot in the middle of the donut, from which the normal emission occurs, can be made as small as 5.8 nm in diameter (Rittweger et al., 2009) but is usually 20 to 50 nm large. The emission depletion light beam can also be formed in an elongated spherical shape to limit the emission to a volume of 45 nm lateral and 108 nm axial resolution (Wildanger et al., 2009). The generation of an image is similar to classical confocal microscopy but due to the very small voxel size, the scanning speed is relatively slow. Improved hardware allowed the reduction of the excitation beam power and the implementation of live imaging. In 2008, Westphal et al. (2008) visualized moving recycling synaptic vesicles labeled with antibody and in the same year the technique was further adapted to visualize fluorescent proteins in living cells (Hein et al., 2008; Nagerl et al., 2008). Other developments made dual color imaging possible enabling colocalization studies (Donnert et al., 2007; Pellett et al., 2011; Tonnesen et al., 2011). STED provides a resolution that is comparable to EM to identify fine structures, such as Bruchpilot, at the active zone of the drosophila neuromuscular junction (Kittel et al., 2006) or dynamic changes of dendritic spines (Nagerl and Bonhoeffer, 2010; Blom et al., 2011; Urban et al., 2011; Tonnesen and Nagerl, 2013b).

## Super-resolution microscopy to study chromaffin cells: present and future

The impact of super-resolution microscopy in the field of neuroendocrinology is just about to spark. STED microscopy has been used to verify that the size of LDCVs was unaltered upon overexpression of a mutated Munc18-1 in neuroendocrine cells (Jorgacevski et al., 2011). SIM was applied to show that a mutated form of synaptobrevin was correctly sorted to LDCVs upon over-expression in mouse chromaffin cells (Borisovska et al., 2012). Furthermore, SIM was used to reveal that the cellular distribution of NPY-mCherry labeled LDCVs was normal and that it was not affected by t-SNARE and Munc18-2 overexpression (Hugo et al., 2013). PALM was used to uncover the size and the shape of clathrin coated pits in PC12 cells during reuptake of vesicular acetylcholine transporters (Sochacki et al., 2012). However, super-resolution microscopy has primarily been used to examine clustering of syntaxin1 and SNAP 25 in the PM. The morphology and the dynamics of syntaxin1 clusters were studied in cracked open PC12 cells using STED (Sieber et al., 2007). In contrast to what was shown using confocal microscopy, Lopez et al. (2009) and Bar-On et al. (2012) used PALM to demonstrate that syntaxin1 and SNAP-25 clusters have a different morphology and that their colocalization is weak in PC12 cells. Additionally, PALM helped to establish that clustered SNARE proteins are not involved in LDCV docking or fusion (Yang et al., 2012). Using a very elegant combination of STED microscopy and Förster resonance energy transfer (FRET) Rickman et al. (2010) showed that overlapping t-SNARE clusters can contain fully assembled t-SNARE acceptor complexes. Finally, PIP2 and PIP3 clustering, that are believed to play a role in the t-SNARE organization, have been investigated using STORM (Wang and Richards, 2012).

Super-resolution microscopy will help us to understand the detailed molecular interactions in chromaffin cells function. As discussed in the introduction, one crucial issue is to clearly demonstrate the correlation between the functional and the morphological data on docking. Another important aspect in chromaffin cell research is to uncover at which time point during the exocytotic process, docking and priming factors bind to the release machinery of LDCVs. FRET is the tool of choice to demonstrate protein interactions in living cells. However, with the remarkable exceptions of Lam et al. (2010) and Zhao et al. (2013), several relatively inconclusive trials using FRET were made to study the interaction of the SNAREs during exocytosis. Thus, it is unlikely that FRET can be used to study the interaction of priming or docking factors with either the LDCVs or the SNARE core complex. One solution to address these problems might be to use a combination of super-resolution microscopy and EM, doing correlative light-electron microscopy (Sjollema et al., 2012). This technique will help us to uncover a relationship between the distance of LDCVs to the plasma membrane and their association with any of these proteins. Finally, aspects of LDCV biogenesis or protein recycling might be better understood using methods such as SIM in combination with molecular manipulations such as gene deletion or protein overexpression.

### Conflict of interest statement

The authors declare that the research was conducted in the absence of any commercial or financial relationships that could be construed as a potential conflict of interest.
